# Supplementary material for: Curated and harmonised transcriptomics datasets of interstitial lung diseases
Source: Data Brief. 2025 Oct 14;63:112139. doi: 10.1016/j.dib.2025.112139 (PMC12581653; doi:10.1016/j.dib.2025.112139)
Supplement: Supplementary file 1 [file mmc1.zip › Supplementary_material/RNA-seq/fastQC reports/GSE124685/SRR8397194_fastqc.html]

SRR8397194.fastq.gz FastQC Report 

FastQC Report

Tue 30 Jan 2024  
SRR8397194.fastq.gz

## Summary

- Basic Statistics
- Per base sequence quality
- Per sequence quality scores
- Per base sequence content
- Per sequence GC content
- Per base N content
- Sequence Length Distribution
- Sequence Duplication Levels
- Overrepresented sequences
- Adapter Content

## Basic Statistics

| Measure | Value |
| --- | --- |
| Filename | SRR8397194.fastq.gz |
| File type | Conventional base calls |
| Encoding | Sanger / Illumina 1.9 |
| Total Sequences | 16895918 |
| Sequences flagged as poor quality | 0 |
| Sequence length | 8-366 |
| %GC | 53 |

## Per base sequence quality

## Per sequence quality scores

## Per base sequence content

## Per sequence GC content

## Per base N content

## Sequence Length Distribution

## Sequence Duplication Levels

## Overrepresented sequences

| Sequence | Count | Percentage | Possible Source |
| --- | --- | --- | --- |
| GGGCCACGAGCTGAGTGCGTCCTGTCACTCCACTCCCATGTCCCTTGGGA | 532849 | 3.153714406047662 | No Hit |
| AAGCTCAGGGAGAGCCCTGTTAGGGCCGCCTCTGGCCCTAGTCTCAGACC | 483362 | 2.860821175860347 | No Hit |
| GGGAATGTCTACGTGCGTATGCACGTGGCACTCTCTGCCCGAGGTCCGGG | 165583 | 0.9800177770749124 | No Hit |
| ACACTGAGGACTCTGTTCCTCCCCTTTCCGCCTAGGGGAAAGTCCCCGGA | 140369 | 0.8307864657013605 | No Hit |
| TCTCTGGGCTTCTATTTCGACCGCGATGATGTGGCTCTGGAAGGCGTGAG | 123810 | 0.732780544981338 | No Hit |
| CTAAGTGCTGTGTTGTCGTTCCCCCTGCTTAAAATAAAGTTGTTTCTTAA | 95354 | 0.5643611669990349 | No Hit |
| GCTGCTACAGGAGAATAGCAGACAGGTATAGTTAAGAAACAACTTTATTT | 92487 | 0.5473925713891368 | No Hit |
| CCACCTTCTCCATCGGCTCCACTGGCCTCGTGGTGTATGACTACCAGCAG | 57662 | 0.34127769796231255 | No Hit |
| AGCGGTTCCGCTGCCCTGAGGCACTCTTCCAGCCTTCCTTCCTGGGCATG | 51025 | 0.30199602057727787 | No Hit |
| AAAAGTGATGGACTGCCCATTGCTGGAGAAGACCTTCTCTCCTACTGTCA | 50648 | 0.29976471239976427 | No Hit |
| TCACACTTCATGATGGAGTTGAAGGTAGTTTCGTGGATGCCACAGGACTC | 50471 | 0.2987171220883056 | No Hit |
| ACTTGATCCCAACTCATCTCTCATTTATTTCGGCTTCTTTTATTCCAGGA | 50085 | 0.2964325466068195 | No Hit |
| ACGCTCGTAGCCCTCGCGCTTCTCCTCGGCCAATTCGCGGAAGAAGTGGC | 49627 | 0.29372183269355356 | No Hit |
| ACGCTCGTAGCCCTCGCGCTTCTCCTCGGCCAGTTCGCGGAAGAAGTGGC | 49265 | 0.291579303355994 | No Hit |
| ACACTGAGGACTCTGTTCCTCCCCTTTCCGCCTAGGGAAAGTCCCCGGAC | 48263 | 0.2856488768470586 | No Hit |
| TGGTTCCTGGCAAGCCCATGTGTGTTGAGAGCTTCTCAGACTATCCACCT | 46775 | 0.2768420159236095 | No Hit |
| TTTACACCTGCTAGGGTGTTCAAAGGTCAGTGCTATAGAAATTCAGTATC | 44854 | 0.26547240582015136 | No Hit |
| ATGATGTAGCAGCAGGTGCCAGGGGCTGGCTTGTAGGCGATCAGCAGCTG | 44141 | 0.2612524516276653 | No Hit |
| ATCAAATCCTGCAGACAAGGGGAGCCCTCAGTCTGCAGGGCTCCATAATG | 44096 | 0.26098611510780295 | No Hit |
| AAAGCCAAGAAAACCAACGATGCCAGATACTGAATTTCTATAGCACTGAC | 44089 | 0.260944684982491 | No Hit |
| ACACCCACCGCAACTGTCTGTCTCATATCACGAACAGCAAAGCGACCCAA | 38286 | 0.22659911109890568 | No Hit |
| GGGCCACGAGCTGAGTGCGTCCTGTCACTCCACTCCCCATGTCCCTTGGG | 30786 | 0.18220969112184376 | No Hit |
| TCTCTGGCTTCTATTTCGACCGCGATGATGTGGCTCTGGAAGGCGTGAGC | 30156 | 0.17848097984377057 | No Hit |
| GGGTACTTCAGGGTCAGGATGCCACGCTTGCTCTGGGCCTCGTCGCCCAC | 26464 | 0.15662954803639553 | No Hit |
| AATAAAAGCGAAAAGAAATGAAAATGTTACACTACATTAATCCTGGAATA | 26246 | 0.15533929556239562 | No Hit |
| ACAAGCAGTTCACAGTAGAACACTGCAGACTGCAGGGCATTTTCAGGCTA | 25862 | 0.15306655725957002 | No Hit |
| GGTTATAATCATTGGCAATGTTGTAATCAACAACCCATGTTATTTCTACA | 25364 | 0.15011909977309312 | No Hit |
| AGCGGTTCCGGTGTCCGGAGGCGCTGTTCCAGCCTTCCTTCCTGGGTATG | 25299 | 0.14973439146662526 | No Hit |
| TGAATCTTGAACTGAGTTCCACTTGTAAACTTCTTGTTTCTTGTGGTTCC | 23969 | 0.1418626676573596 | No Hit |
| TCACACTTCATGATGGAGTTGAAGGTGGTCTCGTGGATGCCGCAAGATTC | 23851 | 0.14116427411638716 | No Hit |
| TGACTGGTGATGCTGATATTGGAGTGCATCTGAGGCTGTTGTAGAAATAA | 22915 | 0.13562447450324983 | No Hit |
| TGTTCCCTCTCCTCATGAGATTGGTGAAGAAAGTATTTGGCAAAGTTCTT | 22422 | 0.1327066099634243 | No Hit |
| TGTCCATGTCTTACTACTTTGACCGCGATGATGTGGCTTTGAAGAACTTT | 20562 | 0.12169803380911294 | No Hit |
| TCGGGCGCCCCAGACACCAGGCGTCATGGTGGGCATGGGCCAGAAGGACT | 20406 | 0.12077473387359006 | No Hit |
| TTGGCTTTCTCTTTCCTCTTCTCCTCCAGGGTGGCTGTCACTGCCTGGTA | 20336 | 0.1203604326204708 | No Hit |
| CTTCCGCATAGCTGCTGTGGTCAAAAAGGAGCCCAGAGTGACAGTTTTCC | 19643 | 0.1162588502145903 | No Hit |
| AGAGGCCACAGTAAGGCCCATCCGAGGTCCTGAGGAGATCTTTTCTCTCT | 18800 | 0.11126947940916854 | No Hit |
| CACTTGGCTTCACCCGTGTGACTTTCGTAACGGGGAGAGAGAGAAAAGAT | 18076 | 0.1069844207340495 | No Hit |
| CAGCTGGAGAAGGACTTCAGCAGCATGAAGAAGTACTGCCAAGTCATCCG | 17976 | 0.106392561801022 | No Hit |
| GGCCTTCTTCTGGCGCAGAGGAAGCAGGCGCATCTGGGTGTGGGCAATGA | 17763 | 0.10513190227367343 | No Hit |
| ATTCTTCCTACCCATGAGCATGGAATGTTCTTCCATTTGTTTGTATCCTC | 17543 | 0.10382981262101296 | No Hit |
| GGAACTCACCTCCCCGAAGCTCAGGGAGAGCCCTGTTAGGGCCGCCTCTG | 17369 | 0.10279997807754512 | No Hit |
| AGAACGGCGACCGTCAAGGAAAACTGTCACTCTGGGCTCCTTTTTGACCACAGCAGCTATGCGGAAGCAGCTGC | 17172 | 0.10163401597948096 | No Hit |
| TCAGAAAGGTCACGTCCTGCTCTTGGTCCTTGTCAGTGCCATGTTCTGTG | 17009 | 0.10066928591864616 | No Hit |

## Adapter Content

Produced by FastQC (version 0.11.7)
